# An experimental study of executive function and social impairment in Cornelia de Lange syndrome

**DOI:** 10.1186/s11689-017-9213-x

**Published:** 2017-09-11

**Authors:** Lisa Nelson, Hayley Crawford, Donna Reid, Joanna Moss, Chris Oliver

**Affiliations:** 1 Cerebra Centre for Neurodevelopmental Disorders, School of Psychology, University of Birmingham, B15 2TT, Edgbaston, UK; 2 Faculty of Health and Life Sciences, Coventry University, Coventry, CV1 5FB UK; 3 Institute of Cognitive Neuroscience, University College London, 17 Queen Square, London, WC1N 3AR UK; 4 Derby Royal Hospital, Uttoxeter Road, Derby, DE22 3NE UK

**Keywords:** Executive function, Social anxiety, Cornelia de Lange syndrome, Down syndrome

## Abstract

**Background:**

Extreme shyness and social anxiety is reported to be characteristic of adolescents and adults with Cornelia de Lange syndrome (CdLS); however, the nature of these characteristics is not well documented. In this study, we develop and apply an experimental assessment of social anxiety in a group of adolescents and adults with CdLS to determine the nature of the social difficulties and whether they are related to impairments in executive functioning.

**Methods:**

A familiar and unfamiliar examiner separately engaged in socially demanding tasks comprising three experimental conditions with a group of individuals with CdLS (*n* = 25; % male = 44; mean age = 22.16; *SD* = 8.81) and a comparable group of individuals with Down syndrome (DS; *n* = 20; % male = 35; mean age = 24.35; *SD* = 5.97). Behaviours indicative of social anxiety were coded. The Behavior Rating Inventory of Executive Function-Preschool version, an informant measure of executive function, was completed by participants’ caregivers.

**Results:**

Significantly less verbalisation was observed in the CdLS group than the DS group in conditions requiring the initiation of speech. In the CdLS group, impairments in verbalisation were not associated with a greater degree of intellectual disability but were significantly correlated with impairments in both planning and working memory. This association was not evident in the DS group.

**Conclusions:**

Adolescents and adults with CdLS have a specific difficulty with the initiation of speech when social demands are placed upon them. This impairment in verbalisation may be underpinned by specific cognitive deficits, although further research is needed to investigate this fully.

## Background

Research has revealed a spectrum of profiles of sociability across genetic syndromes that appears unrelated to degree of intellectual disability. This spectrum includes a heightened level of sociability evident in Angelman, Williams and Down syndromes (DS), and social anxiety in Fragile X (FXS) and Turner syndromes [1–4]. In this study, we aim to identify the nature of aspects of the social impairment of Cornelia de Lange syndrome (CdLS) and the association between social anxiety and executive function impairments.

CdLS affects approximately 1 in 40,000 live births [5] and is associated with intellectual disability as well as specific physical characteristics, including distinctive facial features and limb abnormalities. CdLS is primarily caused by a deletion in the NIPBL gene located on chromosome 5 [6–8] with fewer cases being caused by mutations on the SMC3 gene on chromosome 10 [9], the SMC1A gene [10], the RAD21 gene [11], and the HDAC8 gene [12]. CdLS is associated with mild to profound intellectual disability [13] and a discrepancy between expressive and receptive language skills [13–15].

To date, the social impairment in CdLS has been characterised by social communication difficulties, selective mutism, social anxiety and extreme shyness [16–21]. Our recent research has indicated that individuals with CdLS display less sociability than those with Angelman syndrome, DS and Rubinstein-Taybi syndrome and similar sociability to those with FXS and autism spectrum disorder (ASD), two neurodevelopmental disorders similarly associated with social withdrawal and social anxiety [20]. Children with CdLS have also demonstrated lower levels of social motivation and enjoyment than those with Angelman and Cri du Chat syndromes [22].

Interestingly, both social anxiety and sociability reported in CdLS may be dependent on the demands of the social situation presented. Richards and colleagues [19] investigated the behavioural presentation of social anxiety in children with CdLS compared to children with Cri du Chat syndrome. Although no overall differences emerged on the frequency or duration of behaviours indicative of social anxiety, individuals with CdLS were significantly more likely to display social anxiety-related behaviors immediately before and after eye contact and speech. This suggests that the nature and\or level of social demand may play a role in the presentation of social anxiety in individuals with CdLS. In addition, fine-grained analysis conducted by Moss and colleagues [20] revealed that individuals with CdLS were reported to be more sociable than individuals with FXS and ASD during three out of four social situations with an unfamiliar adult. This research also indicated that individuals with CdLS and other genetic syndromes are significantly more sociable when interacting with a familiar versus unfamiliar adult [20]. The current study aims to further understanding of the social impairment in CdLS by investigating the effect of the familiarity of an interacting adult, and the nature of social demand, on social anxiety-related behaviour.

There is still no ‘gold-standard’ experimental measure of sociability. However, there has been a move towards the experimental assessment of social impairments in the intellectual disability research literature. This has been most notable in the FXS literature (e.g. [23, 24–26]). Several studies on FXS have employed experimental conditions to provide a more detailed picture of social anxiety and the behavioural responses to specific social situations. It has also allowed researchers to determine if there are specific social situations (antecedents) that evoke social anxiety-related behaviours. Some of this research has also investigated differences in social behaviour as a function of both the familiarity of the interacting adult [27] and the examiner’s behaviour [28]. On the basis of this published research, it is clear that experimental methodology involving manipulations of social demand is an effective way to gain a detailed picture of social impairments in individuals who have an intellectual disability. However, careful consideration of the nature of the social tasks is important. One important consideration is the examination of the behaviour of the other person in the interaction which has not been evaluated in the FXS literature on social anxiety. Research in other genetic syndromes, such as DS and Angelman syndromes, has considered the importance of the inter-play between participant and adult behaviour [29–32]. For example, in a study of 13 children with Angelman syndrome, Horsler and colleagues [29] demonstrated that smiling, touch, eye contact and speech from adults were important factors in eliciting smiling and laughing in participants. The present study aims to explore this through behavioural observation of the interacting adult, as well as the participant.

In addition to documenting the phenomenology of social impairment in CdLS, it is also important to consider the cognitive processes that may be associated with the social impairments in this group. Existing literature on a number of neurodevelopmental disorders suggests that specific social impairments are associated with specific executive function processes. The literature on ASD, for example, has generated a wealth of information implicating ‘theory of mind’ deficits in underpinning socio-behavioural impairments characteristic of the disorder [33]. Interestingly, research has demonstrated that theory of mind deficits in FXS are likely to be accounted for by impairments in working memory [34]. More recent research has also identified that specific executive processes may be related to the social impairments reported in ASD. For example, a study examining the association between executive functioning and joint attention impairments in children with ASD found that ventromedial test performance was strongly associated with joint attention skills [35]. These studies demonstrate that social impairments may be subserved by impairments in executive functioning.

In the current study, the relationship between executive functioning and social impairments were examined in order to identify whether impairments in social interactions in CdLS may be associated with specific cognitive impairments. As no gold-standard assessment of social anxiety exists for this population, the study employed novel experimental conditions which manipulate systematically both the *nature of social demand* and the *familiarity of the other person in the interaction*, so these effects on participants’ behaviour, including expressive language, can be examined. The behaviour of the other person in the interaction was also examined. A similar approach has been employed in younger children with CdLS before, highlighting the success of this methodological approach in this population [22]. As DS is associated with a well-delineated phenotype [4, 36, 37], the current study employed a contrast group of individuals with DS to control for the effect of degree of disability and expressive language difficulties. Importantly, chronological age is likely to be an important factor in social impairment. Existing literature indicates an increase in social anxiety and a reduction in sociability with chronological age, with these social impairments being particularly prominent in late adolescence and early adulthood. Therefore, the current study assessed social impairment in adolescents and adults [17, 20].

To summarise, the aims of the current study were to:

1. Investigate whether the familiarity of the interacting adult (hereinafter referred to as examiner) and the nature of social demand impacts differentially on behaviour indicative of social anxiety in adolescents and adults with CdLS and a matched group of participants with DS. It was hypothesised that the CdLS participant group would show more behaviours indicative of social anxiety than the DS group, and that these behaviours would be more prominent in conditions involving an unfamiliar examiner and in conditions with communication demands.
2. Investigate the association between social anxiety and executive function in participants with CdLS, compared to participants with DS. It was hypothesised that compromised executive function would be correlated with social anxiety. Whether or not this would be syndrome-specific was not possible to predict due to limited literature.

## Methods

### Participants

Twenty-five participants with CdLS (11 males and 14 females) aged between 13 and 42 years (mean age = 22.16; *SD* = 8.81) and 20 participants with DS (7 males and 13 females) aged between 15 and 33 years (mean age = 24.35; *SD* = 5.97) took part in this study. Individuals with CdLS were recruited both directly through a research database held at the Cerebra Centre for Neurodevelopmental Disorders, University of Birmingham and indirectly through the CdLS Foundation (UK and Ireland), the parent support group. Participants with DS were recruited through the Cerebra Centre participant database.

The inclusion criteria were as follows: a diagnosis of the relevant syndrome from an appropriate professional, aged 12 years or over, able to speak more than 30 words, mobile, and a self-help score on the Wessex Scale [38] of seven or more (maximum score is 9), indicating that they were able or at the upper end of partly able in terms of self-help skills, or had a receptive vocabulary age equivalent score on the Vineland Adaptive Behavior Scale [VABS; 39] of 40 months or more. Individuals with CdLS who had speech but only used it in certain situations (selective mutism) were still eligible for the study.

A comparison of the group demographics and key characteristics demonstrated that the two groups did not differ significantly in terms of age, gender, receptive language and adaptive behaviour (see Table 1).

**Table 1:** A comparison of demographic information and key characteristics between the Cornelia de Lange and Down syndrome groups. Comparison between participants on: Chronological age, gender, receptive language ability as measured by the British Picture Vocabulary Scale, and adaptive behaviour as measured by the Vineland Adaptive Behavior Scale. Data from the BRIEF-P are also presented here

	CdLS (*n* = 25)	DS (*n* = 20)	*p*
Age (years)			
Mean (SD)	22.16 (8.81)	24.35 (5.97)	.35
Range	13–42	15–33	
Gender			
% Male	44	35	.54
Receptive Language (British Picture Vocabulary Scale)			
Raw score mean (SD)	67.12 (19.96)	69.25 (22.30)	.74
Age equivalence in years mean (SD)	6.16 (2.12)	6.45 (2.68)	.69
Adaptive behaviour (VABS)			
Communication standard score mean (SD)	50.44 (17.58)	50.80 (24.01)	.96
Daily living skills standard score mean (SD)	56.56 (14.18)	57.20 (10.36)	.88
Socialisation domain standard score mean (SD)	57.52 (18.00)	53.40 (25.61)	.59
Adaptive Behavior Composite standard score mean (SD)	54.64 (16.58)	51.33 (18.68)	.56
Executive Function (BRIEF-P)			
Inhibit subscale mean (SD)	26.57 (5.70)	24.37 (4.87)	.192
Shift subscale mean (SD)	19.70 (4.00)	17.11 (4.25)	.049
Emotional control subscale mean (SD)	18.07 (4.47)	15.42 (4.00)	.053
Working memory subscale mean (SD)	29.91 (6.75)	27.44 (6.12)	.234
Plan/organise subscale mean (SD)	17.30 (3.38)	16.16 (3.42)	.283

#### Measures

Parents/primary caregivers of participants completed the following measures:

#### Demographic questionnaire

A demographic questionnaire was used to obtain information regarding participants’ age, gender and diagnostic status (whether a diagnosis had been made and by whom).

#### The Vineland Adaptive Behavior Scale [39]

This semi-structured interview was administered to participant’s parents in order to obtain information regarding participants’ adaptive behaviour skills. There are four domains: Communication, Daily Living Skills, Socialisation, and Motor Skills. Each domain is divided into three further subdomains. An overall Adaptive Behavior Composite may also be derived. Internal consistency ranges from .83–.94 across the domains and .69–.89 across the subdomains.

#### Behavior Rating Inventory of Executive Function-Preschool Version (BRIEF-P; [40])

The BRIEF-P is an informant-based questionnaire used to examine potential deficits in several areas of executive function. The questionnaire consists of 63 items. For each item, the informant rates whether a specific behaviour has been a problem for their child over the previous 6 months using a 3-point Likert scale (never, sometimes, always). The BRIEF-P is made up of five domains: Inhibit, Shift, Emotional Control, Working Memory, Plan/Organise. Higher scores on the BRIEF-P are suggestive of greater perceived deficits. The psychometric properties of the BRIEF-P appear robust. Studies have demonstrated that the measure captures profiles of executive functioning that differ across various disorders, including attention-deficit hyperactivity disorder and ASD [41]. Although the BRIEF-P was designed for individuals who are younger than the participants in the current study, it was deemed a more appropriate measure than the BRIEF (5–18 years) based on the suitability of the items for the participant’s level of intellectual disability. An informant measure of executive function was used in the current study, rather than a performance-based measure. Informant-based measures have been described to tap into how participants interpret and react to a situation without being directed to perform a specific task or being taught a rule [42]. This suggests that informant measures, such as the BRIEF-P, capture participant’s everyday executive function skills, as opposed to best possible performance on a task, which was deemed important for the current study.

#### The British Picture Vocabulary Scale—second edition (BPVS-II; [43])

The BPVS-II was used to assess receptive vocabulary. The assessment comprises 168 items. The administration of the test allows basal and ceiling levels to be established without needing to administer the entire test. For each item, the participant is required to select one of four pictures from a stimulus booklet that most accurately represents the meaning of the word spoken by the examiner. The test has been standardised on typically developing individuals and it has been reported to be psychometrically robust with good validity and reliability.

#### Social Tasks

The Social Tasks were designed to assess whether behaviours indicative of social anxiety are evoked by various social situations. The Social Tasks comprised one control condition and three experimental conditions. The experimental conditions are designed to place increasing social demands upon the participant. The experimental conditions are *Voluntary Social Interaction*, *Required Social Interaction* and *Performance*. They were administered as follows:

1. The control condition is a modified version of the ‘Break’ condition from modules three and four of the Autism Diagnostic Observation Schedule (ADOS; [44]). During this condition, the participant and examiner are sat at a table. The participant is given some items (paper and pens, newspaper, magazine and some puzzles) to engage with, whilst the examiner either does some work or reads a magazine. The examiner is still in close proximity to the participant during this condition to control for the presence of the examiner in the experimental conditions. The control condition lasts for approximately 4 min.
2. The Voluntary Social Interaction condition involves the examiner showing the participant a series of 20 holiday photographs and making pre-determined comments about every other photograph. Here, the participant is provided with the opportunity to make a comment about the photographs or respond to a comment made by the examiner, but there is no explicit expectation for them to do so. This condition is not timed and finishes after the last photograph has been shown to the participant.
3. The Required Social Interaction condition involves a conversation between the examiner and participant, whereby the examiner asks the participant a series of questions and the participant *is* explicitly expected to respond to them. The conversation also provides the participant with the opportunity to initiate conversation with the examiner by asking the examiner questions. The examiner predominantly leads this condition because they ask the participant questions in order to maintain the conversation. The Required Social Interaction condition lasts for approximately 4 min.
4. The Performance condition is a modified version of the ‘Cartoons’ condition from the ADOS [44] and utilises both sets of cartoons from the ADOS. The examiner tells the participant the story in one of the cartoons and then asks the participant to stand up and tell them the story back. This procedure is then repeated for a second cartoon. The participants are expected to stand up and re-tell or ‘perform’ a story on their own without guidance. Only if the participant shows difficulty with retelling the story does the examiner prompt. This condition is not timed and finishes after the participant has presented both cartoons.

A familiar examiner and an unfamiliar examiner carried out the four conditions separately, in order to identify whether there was an effect of familiarity on the Social Tasks. The familiar examiner was someone the participant sees at least three times a week, e.g. their main caregiver, their teacher, their support worker, etc. The unfamiliar examiner was a trained confederate involved in the project who had never met the participant. The order of conditions and whether the familiar examiner or unfamiliar examiner administered the conditions first were counterbalanced.

#### Real-time coding of social tasks

The literature on observational indicators of social anxiety in both typically developing children and individuals with intellectual disabilities was examined to identify indicators of social anxiety [23–25, 27, 45–51]. Behaviours previously identified in existing literature as indicators of social anxiety were coded during each condition of the Social Tasks. Several examiner behaviours are also coded during the conditions and used in the analysis to provide a more detailed picture of the nature of the interaction between the examiner and the participant. All behaviours are operationally defined. Table 2 shows all the behaviours that were included in the analysis. Behaviours were coded using Obswin 3.2 [52]. The Voluntary Social Interaction and Performance conditions were coded for the full length of time that they had been recorded for because these conditions were dependent on other factors, i.e. the Voluntary Social Interaction condition finished once all 20 photographs had been shown to the participant and the Performance condition finished once the participant had explained the story in both cartoons. The first 4 min of the control condition and the Required Social Interaction condition were coded so that the duration of these conditions were matched across the groups. Some behaviours were coded as durations (i.e. behaviours with an onset and an offset) and some were coded as events (i.e. behaviours of such short duration that only their occurrence is recorded)*.* Table 2 shows whether behaviours were coded as events or durations.

**Table 2:** Operationalised definitions of behaviours coded as control variables; and participant and examiner behaviours used in the analysis

Behaviour	Operationalised definitions
Participant verbalisation	
*Participant verbalisation* (duration)	The participant’s speech; These may be utterances (e.g. ‘erm’), words, phrases or sentences. The person may use speech for the purpose of communication with someone else, e.g. asking a question, making a comment, answering a question or the speech may be used when the person is talking to himself or herself. The participant’s speech may be intelligible or unintelligible.
*Participant question* (event)	The participant asks the examiner a question. For example, ‘Did you drive here?’
*Participant offers information* (event)	The participant spontaneously (not in response to a question) offers information. The information may or may not be about them. For example, ‘I went to the beach on holiday’ or ‘the cartoon is funny’.
*Participant verbal response* (event)	The participant responds verbally to a question, statement, comment, prompt or request made by the examiner by providing information. N.b. this code also includes the participant’s description of the cartoons in the Cartoon condition.
Participant non-verbal behaviour	
*Participant positive facial expression* (duration)	The participant demonstrates a positive facial expression, for example, laughing or smiling. Facial expression must clearly indicate expression of pleasure in activity or conversation. Facial expression may or may not be directed towards the examiner.
*Participant looks at examiner* (duration)	The participant looks in the direction of the examiner’s eyes or face.
*Participant nod/shake* (event)	The participant responds to a question, statement, comment or prompt made by the examiner, by nodding their head to indicate ‘yes’ or shaking their head to indicate ‘no’. This *does not* include use of Makaton or British Sign Language.
*Participant descriptive gestures* (duration)	The participant uses movements of their arms or hands to help them describe something.
*Participant fidget* (duration)	The participant displays restless, repetitive, non-rhythmic, non-functional motor movements, such as, moving their hands, touching their face or hair or moving an object, or wriggling in their seat. This code *does not* include stereotyped behaviours, which are *rhythmic*, unusual seemingly purposeless movements of their body or objects (based on Lesniak-Karpiak, Mazzocco & Ross, 2003 [23]).
Examiner verbalisation	
*Examiner verbalisation* (duration)	The examiner’s speech; These may be utterances (e.g. ‘erm’), words, phrases or sentences. The person may use speech for the purpose of communication with someone else, e.g. asking a question, making a comment, answering a question or the speech may be used when the person is talking to himself or herself. The examiner’s speech may be intelligible or unintelligible.
*Examiner question* (event)	The examiner asks the participant a question, which requires a response from the participant. For example’ What books do you like?’
*Examiner prompt* (event)	The examiner prompts the participant to respond by repeating or slightly paraphrasing the original question, request, comment or piece of information.
*Examiner verbal response* (event)	The examiner responds to the participant’s verbal question, comment, statement or offering of information using verbal communication to give the appropriate information.
*Examiner Offers information* (event)	The examiner spontaneously (not in response to a question) offers information. The information may or may not be about themselves. For example ‘I came from Birmingham’. N.b. this code also includes the examiner’s description of the cartoons in the Cartoon condition.
Behaviours coded as control variables	
*Participant engage with task* (duration)	The participant looks at and/or touches an object allocated for a condition. This may be reading a magazine / newspaper, colouring with felt tips, listening to the radio in the ‘Break’ condition; looking at or touching the photographs in the ‘Photograph’ condition; looking at or touching the cartoon in the ‘Cartoon’ condition. Objects that have not been incorporated as part of the social presses *should not* be coded, e.g. if the person is drinking from a cup or mug, which is on the table. This code *does not* apply to the ‘Conversation’ condition because no objects are required for this condition.
*Examiner looks at participant* (duration)	The examiner is looking in the direction of the participant’s eyes or face.

The following three variables were coded in addition to the participant and examiner outcome behaviours: examiner off camera, participant off camera and participant’s hands off camera. These variables affected whether several outcome variables could be coded during a condition, e.g. if the participant was off camera, then ‘participant looks at examiner’ could not be coded. For the purpose of calculating more accurate durations and frequencies of outcome behaviours, if any of these three variables occurred for 10% or more of the time in a condition then the outcome behaviours affected by these variables were recalculated to only take into account the time when these behaviours could be coded, e.g. if a participant’s hands were off the camera for 15% of time during a condition, then ‘participant fidgets’ was only coded during the 85% of time during which the participant’s hands could be seen.

Inter-rater reliability was conducted on all behaviours coded in the Social Tasks for 26.67% of participants (25% of Down syndrome participants and 28% of CdLS participants). Agreement between two independent raters was calculated using Cohen’s Kappa co-efficient based on 5-s interval-by-interval basis. The mean level of agreement across the participant behaviours was .64 (range .48 to .82). The mean level of agreement across the examiner behaviours was .59 (range .44 to .85). This reliability was considered to be moderate—very good [53].

#### Procedure

All participants were visited at their home. The first assessment to be conducted on all research visits was the Social Tasks so that the researcher acting as the unfamiliar examiner would have had minimal contact with the participant. The Social Tasks were always conducted in a room with a table and only the participant and familiar or unfamiliar examiner were present. The Social Task conditions were counterbalanced so that there were no order effects across the groups.

After the Social Tasks were completed, the BPVS-II [43] was administered. The VABS-II [39] was administered to either the participant’s main caregiver or key worker at a convenient time for them, during the research visit day. After the research visits had taken place, footage from the Social Tasks was coded.

### Data analysis

A preliminary analysis was conducted to ensure that the Social Tasks were administered uniformly across groups. The duration of the condition, the duration of the participant engaging in the task and the duration of the examiner looking at the participant were examined. See Table 3 for differences on these variables. The majority of differences were not significant (*p* < .05). The differences that were significant were marginal differences which could not be controlled for given the need to keep the conditions as representative of naturalistic social situations as possible. These analyses show that any significant differences identified between the groups in any of the behavioural outcome variables are not due to differences in the administration of the Social Tasks.

**Table 3:** Differences between the Cornelia de Lange syndrome and Down syndrome groups on control variables

Behaviour	Condition	CdLS median (IQR)	DS median (IQR)	*U*	*Z*	*p*
Duration of condition	Familiar voluntary social interaction	341 (361.50)	355 (200.00)	236.5	−.02	.98
	Unfamiliar voluntary social interaction	225 (105.50)	228.5 (84.25)	217	−.75	.45
	Familiar required social interaction	240 (.50)	240 (6.00)	218	−.59	.56
	Unfamiliar required social interaction	240 (.00)	240 (0.00)	236	−.74	.46
	Familiar performance	151 (161.00)	109 (121.00)	173	−1.53	.13
	Unfamiliar performance	142 (112.00)	125 (62.00)	150.5	−2.06	*<.05*
Participant engage in task	Familiar voluntary social interaction	89.67 (25.44)	97.97 (5.23)	89	−3.52	*<.001*
	Unfamiliar voluntary social interaction	96.15 (19.95)	96.63 (5.68)	180.5	−1.59	.11
	Familiar required social interaction	N/A				
	Unfamiliar required social interaction	N/A				
	Familiar performance	87.5 (17.39)	97.16 (11.33)	145.5	−2.18	*<.05*
	Unfamiliar performance	92.91 (27.41)	95.49 (4.72)	192.5	−1.07	.29
Examiner looks at participant	Familiar voluntary social interaction	27.05 (19.77)	22.04 (13.44)	186	−1.22	.22
	Unfamiliar voluntary social interaction	21.36 (28.20)	37.27 (18.17)	134	−2.65	*<.01*
	Familiar required social interaction	94.58 (18.34)	97.5 (14.59)	181	−1.34	.18
	Unfamiliar required social interaction	95.83 (10.63)	94.79 (7.40)	248.5	−.03	.97
	Familiar performance	0 (5.80)	0 (28.30)	233	−.14	.89
	Unfamiliar performance	0 (43.44)	0 (0.00)	149	−2.48	*<.05*

*N/A* not applicable

The data for almost all the outcome variables were not normally distributed across all conditions (Kolmogrov-Smirnov test*; p* < .05) and consequently non-parametric tests were employed throughout the analysis. The analyses examined the effect of group (CdLS, DS), nature of demand (Voluntary Social Interaction, Required Social Interaction, Performance) and familiarity (unfamiliar examiner, familiar examiner) on the outcome variables. Participant outcome variables included verbal behaviours (verbalisation, question-asking, offering of information, and responses) and non-verbal behaviours (positive facial expression, looking to the examiner, nodding/shaking head, gestures, and fidgeting). Examiner outcome variables included verbal behaviour (verbalisation, question-asking, prompting, responses, and offering of information). See Table 2 for operationalised definitions of each outcome variable.

## Results

### Preliminary analysis: comparison between the control condition and experimental conditions

An analysis was conducted initially for each group to ensure that participant outcome variables examined in the experimental conditions were evoked by social demands. Consequently, pairwise Wilcoxon rank sum tests were conducted separately for each group to compare each participant outcome variable between the control condition and each of the experimental conditions.^1^ All but one^2^ of the analyses were significant with all the outcome variables being observed for significantly longer in the experimental conditions than the control condition, demonstrating that the outcome variables being examined in the current study were evoked by the social demands of the experimental conditions.

### Comparison of outcome variables on social tasks

#### Participant behaviour

**Fig. 1:**
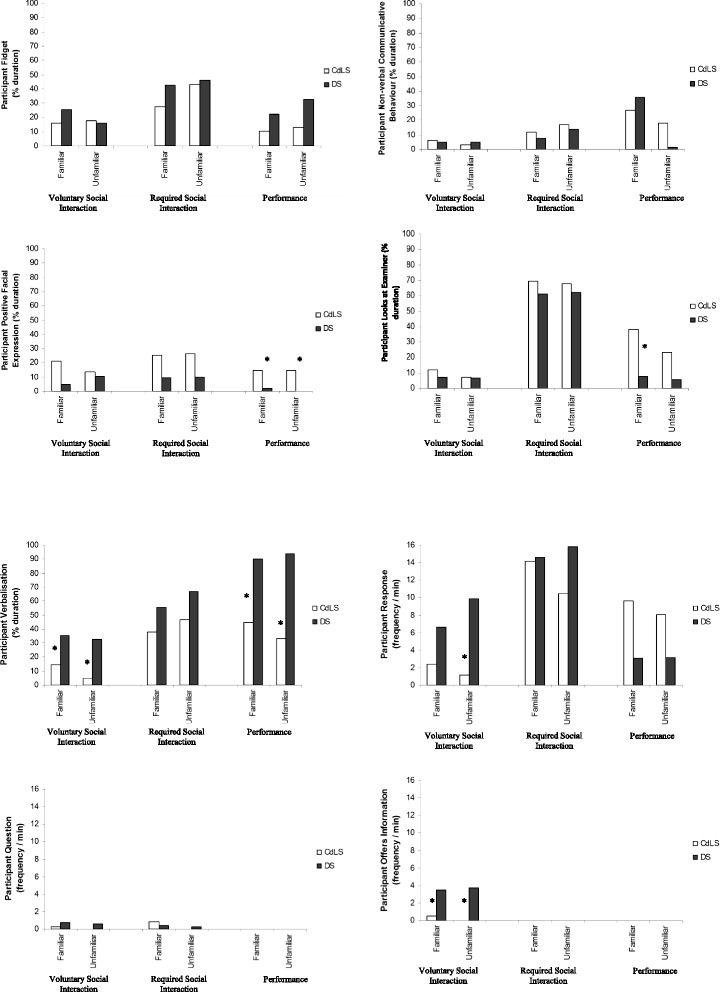
Participant outcome variables for the Down syndrome and Cornelia de Lange syndrome groups; *asterisk* indicates significant between-groups difference (*p* < .005)

Figure 1 shows median duration/frequency of the participant outcome variables for both the CdLS and DS groups. A more conservative alpha level (*p* < .005) was employed for this set of analyses.

The analysis revealed a two-way interaction between group and nature of demand for participant verbalisation. The CdLS group showed significantly less verbalisation than the DS group in both the familiar and unfamiliar Voluntary Social Interaction conditions (*U* = 108, *p* < .005; *U* = 65, *p* < .001) and both the familiar and unfamiliar Performance conditions (*U* = 77.5, *p* < .001; *U* = 109, *p* < .005). The difference in verbalisation between the groups in the unfamiliar Required Social Interaction condition approached significance (*p* = .006).

An analysis of the type of participant verbalisation shown in the Voluntary Social Interaction and Performance conditions revealed that there was a significant difference between the groups in the type of verbalisation shown in the Voluntary Social Interaction condition only. The DS group demonstrated significantly more offering of information than the CdLS group in both the familiar and unfamiliar Voluntary Social Interaction conditions (*U* = 62, *p* < .001; *U* = 46, *p* < .001) and also responded significantly more often than the CdLS group in the unfamiliar Voluntary Social Interaction condition (*U* = 73, *p* < .001). The analyses also revealed a main effect of familiarity for participant verbalisation in the Required Social Interaction condition for the DS group. Interestingly, the DS group actually showed significantly more verbalisation in the unfamiliar Required Social Interaction condition than in the familiar Required Social Interaction condition (*z* = −3.14, *p* < .005).

Surprisingly, the analysis also showed significantly more positive facial expression by the CdLS group in comparison to the DS group in the familiar and unfamiliar Performance conditions (*U* = 113, *p* < .005; *U* = 75, *p* < .001). The analysis also revealed that the CdLS group looked at the examiner for a significantly longer duration than the DS group in the familiar Performance condition (*U* = 99, *p* = .001). Finally, the analysis demonstrated that there was no significant difference in fidgeting or non-verbal communicative behaviour between the two groups, in any condition.

#### Examiner behaviour

**Fig. 2:**
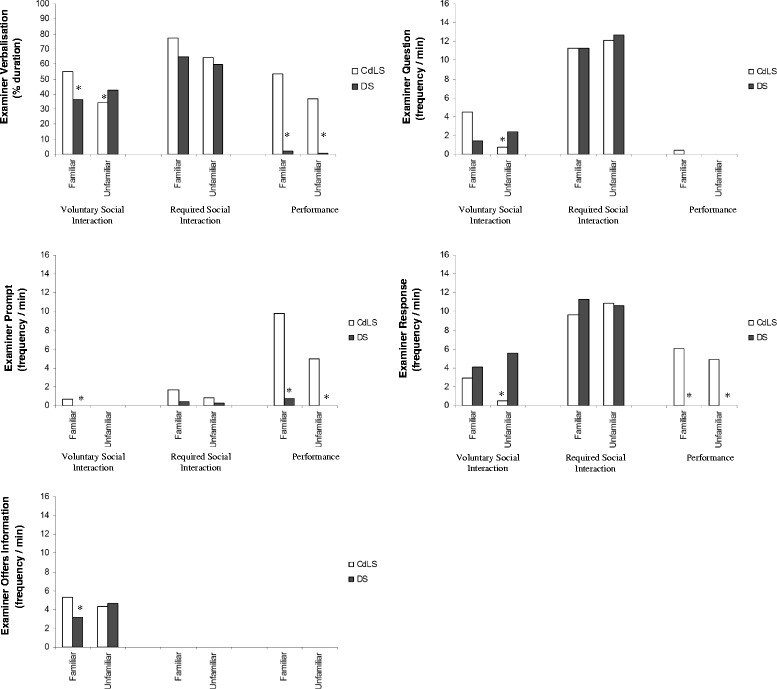
Examiner outcome variables for the Down syndrome and Cornelia de Lange syndrome groups; *asterisk* indicates significant between-groups difference (*p* < .005)

Figure 2 shows the median duration/ frequency of the examiner outcome variables for both the CdLS and DS groups. An analysis of examiner verbalisation revealed a two-way interaction between group and nature of demand as significant differences were found between the two groups in the Voluntary Social Interaction condition and the Performance condition, but not in the Required Social Interaction condition. Significantly more verbalisation was shown by the familiar and unfamiliar examiners when interacting with the CdLS group in the Performance condition when compared to the DS group (*U* = 23, *p* < .001; *U* = 43, *p* < .001). In the Voluntary Social Interaction conditions, significantly more verbalisation was also shown by the familiar examiners with the CdLS group in comparison to the DS group (*U* = 112, *p* < .005). The unfamiliar examiners, however, showed significantly more verbalisation with the DS participants than the CdLS participants in this condition (*U* = 119, *p* < .005).

An analysis of the type of examiner verbalisation shown in the Voluntary Social Interaction and Performance conditions revealed that familiar and unfamiliar examiners used significantly more prompts (*U* = 70, *p* < .001; *U* = 85.5, *p* < .001) and responses (*U* = 32, *p* < .001; *U* = 67, *p* < .001) with the CdLS group than the DS group in the Performance condition. In the Voluntary Social Interaction conditions, the familiar examiners gave significantly more prompts (*U* = 107.5, *p* < .005) and offering of information (*U* = 100, *p* < .005) to the CdLS group than the DS group, whilst the unfamiliar examiners gave significantly more questions (*U* = 106, *p* < .005) and responses (*U* = 97, *p* < .001) to the DS group than the CdLS group.

### Association between social impairments and cognitive functioning in Cornelia de Lange syndrome

As the current study has identified a specific impairment in verbalisation for the CdLS group, this was correlated with a measure of executive functioning. For the purpose of this analysis, the mean duration of participant verbalisation across the familiar and unfamiliar Performance conditions was used for examining the relationship between verbalisation and executive functioning because this condition placed the highest social (and thus cognitive) demands on participants. In addition, mean participant verbalisation across the Performance conditions was correlated with age, receptive and expressive language and adaptive behaviour in order to examine whether these broader developmental variables were also related to verbalisation in either group. A series of Spearman’s correlations were conducted for this analysis. Table 4 shows the results for these correlations.

**Table 4:** Correlations between mean participant verbalisation across the Performance conditions and age, receptive and expressive language and adaptive behaviour for the Cornelia de Lange syndrome and Down syndrome groups

	CdLS mean participant verbalisation	DS mean participant verbalisation
Chronological age (years)	.30	.16
BPVS raw score	.53**	.81**
VABS communication domain standard score	−.16	.76**
VABS daily living skills domain standard score	.30	.75**
VABS socialisation domain standard score	.27	.59*

*p < .05

**p < .01

The analysis revealed that only receptive language (measured by the BPVS) was significantly, positively correlated with verbalisation in the CdLS group. In the DS group, both language and adaptive behaviour were significantly correlated with verbalisation. The pattern of correlations observed for the DS group was expected given that as verbalisation increases, adaptive behaviour would also be expected to increase. The dissociation of the relationship between verbalisation and adaptive behaviour in the CdLS group suggested that these individuals may have a specific cognitive impairment that is related to language and was independent of global development of adaptive behaviour.

Table 5 shows the correlations between mean verbalisation and the BRIEF-P subscales for the CdLS and DS groups. A series of Spearman’s correlations^3^ between mean verbalisation across the Performance conditions and subscale scores on the BRIEF-P revealed that there were significant associations between the duration of verbalisation and working memory, and the duration of verbalisation and planning in the CdLS group but these associations were not evident in the DS group. The correlation between the Inhibit subscale and verbalisation approached significance in the CdLS group. No significant correlations between any of the BRIEF-P subscales and the duration of verbalisation was found in the DS group. The significant correlations found for the CdLS group indicate that less verbalisation in the Performance condition was associated with poorer performance on working memory and planning assessments.

**Table 5:** Correlations between mean participant verbalisation across the Performance conditions and BRIEF-P subscales for both the Cornelia de Lange syndrome and Down syndrome groups

BRIEF-P subscale	CdLS mean participant verbalisation	DS mean participant verbalisation
Inhibit	.41	−.24
Shift	−.26	.07
Emotional control	−.31	−.12
Working memory	−.57**	.10
Plan/organise	−.62**	−.07

*p < .05

**p < .01

## Discussion

This novel experimental study assessed the phenomenology of the social impairment in verbal adolescents and adults with CdLS in contrast to a group of adolescents and adults with DS. This is the first study on social anxiety in CdLS to employ a robust factorial, experimental design, placing different social demands on participants whilst varying familiarity, in order to examine which factors evoked behaviours indicative of social anxiety. The study examined the relationship between any social impairments identified in the CdLS group and cognitive functioning in order to identify whether there was preliminary evidence for specific cognitive impairments underpinning specific social impairments in this group.

The most striking difference identified between the two groups was in the duration of participant verbalisation. The CdLS group showed significantly less verbalisation than the DS group in the familiar and unfamiliar Voluntary Social Interaction and Performance conditions, whilst no significant group difference was observed in the Required Social Interaction condition where there was an explicit expectation to verbalise. It appears that there are specific social demands in the Voluntary Social Interaction and Performance conditions which reduce verbalisation in the CdLS group. The two conditions which showed group differences in verbalisation rely more heavily on participants being able to initiate speech, so it may be that this is a particular difficulty for the CdLS group. For example, verbalisation in the Voluntary Social Interaction condition relies on participants being able to initiate speech to comment (offering information) on photographs or respond to a comment made by the examiner on a photograph (response), and there is no explicit expectation for the participant to do this. Taken together, these findings suggest that individuals with CdLS have a specific difficulty with the initiation of speech, particularly when the expectation to do so is implicit, which results in a marked reduction in verbalisation when social demands involving the initiation of speech are placed upon individuals with CdLS. Interestingly, participants with CdLS also looked at the examiner for longer than the DS group, indicating that participants with CdLS are not demonstrating complete social withdrawal, but rather the lack of social motivation is specific to verbalisation. It is unlikely that these differences in verbalisation were a product of expressive language deficits in the CdLS as the two participant groups did not differ on the Expressive Language Subdomain of the VABS. Although not a direct measure of expressive language, this measure, completed by parents, is more likely to reflect the abilities of participants with CdLS due to the elevated rates of selective mutism in this population. However, future research should examine this further to disentangle the effects of expressive language abilities on verbalisation in social situations which differ in terms of expectation of verbalisation.

This is the first empirical evidence showing a reduction in speech, in adolescents and adults with CdLS that may be due to a specific difficulty in the initiation of speech. These findings contribute to the sparse literature on social impairments in CdLS. To date, only one study has been published on the phenomenology of social anxiety in CdLS and this study found no significant difference in communication, which included both verbal and non-verbal communication, between children with CdLS and children in a comparable contrast group [19]. Although these findings do appear to contrast with results reported in the current study, where we report significantly less verbalisation in individuals with CdLS compared to those with DS, these differences were particularly prominent in the Voluntary Social Interaction and Performance conditions. Interestingly, Richards and colleagues [19] reported that individuals with CdLS were significantly more likely to display social-anxiety related behaviours immediately before and after eye contact and speech, suggesting that social anxiety is heightened in CdLS, particularly at the point of speech initiation. The consistency of findings indicating that social anxiety in CdLS is mediated by the type of social situation is particularly interesting given the different ages of participants across samples. Specifically, the mean age of participants in the current study was 22 years, whereas the mean age of participants in Richards et al. [19] was 11 years. Socio-behavioural characteristics have been reported to change with age in CdLS, such that social anxiety increases and sociability decreases during early adulthood [17, 20].

The findings in the current study therefore indicate that specific social demands reduce verbalisation in individuals with CdLS, with the familiarity of the other person being relatively unimportant. However, more in-depth analysis regarding the type of verbalisation revealed some empirical evidence for the effect of familiarity in the Voluntary Social Interaction condition. Specifically, the CdLS group responded significantly less to comments made by the unfamiliar examiner than the DS group, yet no significant difference was found between the groups in responding to the familiar examiner in this condition. This suggests that the presence of an unfamiliar examiner caused a significant reduction in responses by the CdLS group, providing support for the effect of familiarity on social interactions in CdLS. These results support previous literature indicating that the familiarity of the other person in the interaction does affect sociability in adolescents and adults with CdLS [17, 20, 54].

Interestingly, no significant differences were found between the groups on some additional indicators of social anxiety such as fidgeting and non-verbal behaviour. In addition, the CdLS group actually showed significantly more positive facial expression with the familiar and unfamiliar examiners in the Performance condition and looked significantly longer at the familiar examiner in the Performance condition than the DS group. These are unexpected findings given that a longer duration of positive facial expression and a longer duration of looking in the direction of the examiner would not be expected if social anxiety was evident in the CdLS group. This supports the notion that the lack of social motivation or engagement in individuals with CdLS is specific to verbalisation and is not reflective of more global social withdrawal. It is likely that a specific communication problem affecting the initiation of speech makes it appear that individuals with CdLS show anxiety in social situations. Although this is possible, the reported effect of the presence of unfamiliar people on levels of sociability in the literature for individuals with CdLS would suggest that there is some anxiety-related difficulty in this group. Therefore, it may be that there is a communication problem which is enhanced by anxiety caused by the presence of unfamiliar people. A positive facial expression and looking in the direction of the examiner may then serve to compensate for the lack of verbalisation in demanding conditions or act as a coping strategy, prompting the examiner to speak on their behalf. This is supported by the fact that these behaviours were shown in the Performance condition where the most difficulties in verbalisation were evident.

Group differences were found in the duration of examiner verbalisation in the Voluntary Social Interaction and Performance conditions. Familiar and unfamiliar examiners showed significantly more verbalisation in the Performance condition with significantly more prompts and responses being used for the CdLS participants compared to the DS participants. It appears that the examiners tried to help the CdLS participants, although, this increase in verbalisation by examiners may have further increased the demands on the CdLS participants. The familiar examiners in the Voluntary Social Interaction condition also demonstrated this pattern of behaviour as familiar examiners used significantly more comments and prompts with the CdLS group. Interestingly, the Voluntary Social Interaction condition does not involve examiners prompting participants because there is no explicit expectation for participants to verbalise. Perhaps this indicates that the familiar examiners will try to prompt individuals with CdLS to verbalise whenever they can to encourage individuals to verbalise. This research indicates that further exploration of the extent to which participant social behaviour is governed by examiner behaviour is warranted.

There is currently no study of CdLS that examines how participant and examiner behaviour affect one another in social interactions. Therefore, this is the first study to contribute to the literature in this way. Further research examining the inter-play between participant and examiner behaviours would be useful to determine how these may affect one another. Research in other genetic syndromes has already demonstrated the inter-play between participant and adult behaviour. For example, increased laughing and smiling by individuals with Angelman syndrome is evoked by increased social interactions with adults and increased social contact from adults [1]. This type of research is important in CdLS because it may also help when devising intervention strategies, e.g. asking adults not to prompt the person if it increases further demands on them.

In addition to describing the social impairment in CdLS, the current study also examined whether social impairments observed in the CdLS group were related to specific cognitive impairments. The results indicated that reduction of verbalisations in the CdLS group was associated with impairments in both planning and working memory. This was further supported by the fact that this relationship was not evident in the DS group and the fact that verbalisation was not related to adaptive behaviour in the CdLS group. It cannot be assumed that the relationship between verbalisation and cognitive impairments is causal from the correlational analysis and the use of an informant-based measure of executive functioning. However, the fact that a significant association between these domains was present in the CdLS group, but not in the DS group, suggests that further investigations examining the relationship between planning, working memory and verbalisation in CdLS are needed to understand whether deficits in working memory and planning underpin the difficulties observed in verbalisation in this group. Interestingly, whilst the relationship between inhibition and verbalisation approached significance, verbalisation was not related to the inhibition and attention switching in the same way. One interpretation of these findings concerns the reliance on working memory and planning resources in a social exchange with regard to holding conversational information in mind, and planning a response. Inhibition may similarly be required to restrict prepotent verbal responses; however, attention shifting and emotional control may not be relied upon to the same extent for the verbalisation aspect of a social exchange.

There were several limitations to the current study that may affect the interpretation of the findings. Only behavioural indicators of social anxiety were employed in the current study which meant that it was difficult to fully determine whether a reduction in verbalisation in the CdLS group was due to or affected by anxiety caused by the presence of unfamiliar people. Physiological measures have been used in combination with behavioural indicators of social anxiety in the FXS literature [24, 25] to provide a more accurate picture about whether the behaviours shown in this group are anxiety-related. Any future research on social anxiety in CdLS should try to incorporate physiological measures as well as behavioural indicators. Furthermore, although preliminary analyses indicate differences in social behaviour between the control condition and experimental conditions, which points to the integrity of the Social Tasks, validation of the measure in a typically developing population would further demonstrate that the conditions differed in social pressure. An additional limitation to the present study is the lack of information available about any anti-anxiety medication that participants may have been taking at the time of data collection. Another drawback is that the levels of social anxiety in adolescents and adults with CdLS may be under-reported. Two individuals with CdLS were recruited for the current study but withdrew before the research visits because parents reported that both individuals were experiencing significant anxiety about being visited by an unfamiliar person. The fact that these and other individuals with CdLS may not have taken part in the current study due to anxiety about being visited by an unfamiliar person indicates that the effect of unfamiliar people on levels of anxiety may be under-reported in this study.

## Conclusions

Despite the limitations, this study has still provided several important findings that contribute to the literature on social impairments in CdLS. The results suggest that adolescents and adults with CdLS have a specific difficulty with the initiation of speech that leads to a reduction in verbalisation when social demands involving the initiation of speech are placed upon individuals. Although, the evidence was not conclusive in the current study, adolescents and adults with CdLS seem to show increased anxiety in the presence of unfamiliar people which causes a further reduction in speech. The results from the current study also indicate that there is a syndrome-environment interaction between verbalisation in adolescents and adults with CdLS and verbalisation in examiners interacting with them. It seems that a reduction in verbalisation in adolescents and adults with CdLS is related to increased verbalisation in examiners interacting with them. It may be that this increased examiner verbalisation causes further demands on verbalisation in people with CdLS and increases the cognitive and social demand. The study also provided some preliminary evidence for a relationship between verbalisation, working memory and planning in CdLS. Research is needed to examine the pathway from cognition to behaviour in CdLS in order to identify the cause of the verbal impairment identified in this study and use this to develop helpful prevention and intervention strategies. Furthermore, a clearer understanding of the association between anxiety and verbalisation in this group is needed to understand how these factors impact upon each other.

## Abbreviations

ADOS: Autism Diagnostic Observation Schedule
ASD: Autism spectrum disorder
BPVS: British Picture Vocabulary Scale
BRI: Behavioural Regulation Index
BRIEF-P: Behaviour Rating Inventory of Executive Function-Preschool Version
CdLS: Cornelia de Lange syndrome
DS: Down syndrome
FXS: Fragile X syndrome
GEC: Global Executive Function Composite
MI: Metacognition Index
VABS: Vineland Adaptive Behavior Scale

## Footnotes

1 A mean score was taken across the three experimental conditions.

2 A significant difference was not found in positive facial expression (*p* = .02) for the DS group between the familiar control condition and the mean of the experimental conditions. This was due to the low level of positive facial expression shown by the group in the experimental conditions.

3 Pearson’s Partial correlations between mean participant verbalisation and the BRIEF-P subscales, whilst controlling for BPVS scores in the CdLS group and BPVS and VABS in the DS group, showed the same findings as the Pearson’s correlations.
